# Clinico-pathology, diagnosis and management of *Cysticercus fasciolaris* and *Hymenolepis diminuta* co-infection in wistar rats

**DOI:** 10.14202/vetworld.2015.116-120

**Published:** 2015-01-29

**Authors:** Y. Damodar Singh, Rahul Singh Arya

**Affiliations:** Department of Veterinary Pathology, College of Veterinary Sciences & Animal Husbandry, Central Agricultural University, Selesih, Aizawl - 796014, Mizoram, India

**Keywords:** anthelmintics, co-infection, *Hymenolepis diminuta*, Mizoram, *Taenia taeniaeformis*, wistar rats

## Abstract

**Aim:**

The present study was undertaken to study the pathology and control of sudden unexplained mortality in wistar rats.

**Materials and Methods:**

This study was conducted in a colony of 25 male wistar rats where there was mortality of nine rats. The dead rats were subjected to thorough post-mortem examination and necropsy samples were processed for hematoxylin and eosin staining for histopathological studies. Faecal samples of live rats were studied for the presence of parasitic eggs. Treatment with anthelmintics was given to manage the mortality and infections.

**Results:**

The investigation revealed a natural co-infection of *Cysticercus fasciolaris* and *Hymenolepis diminuta* in wistar rats, which were pathogenic enough to cause mortality. Typical lesions associated with the parasites were found in the dead rats. The mortality and infection were managed with common anthelmintics.

**Conclusion:**

*C. fasciolaris* and *H. diminuta* infection can cause mortality in wistar rats even when individually they cause asymptomatic infection. The mortality and infection can be managed with common anthelmintics.

## Introduction

*Taenia taeniaeformis* occurs all over the world in small intestines of their definitive host, cats and related carnivores. The infected definitive host voids thousands of eggs daily which then infect the intermediate host through contamination of feed and water. These include rodents and less frequently lagomorphs. Its metacestode, a *strobilocercus* (*Cysticercus fasciolaris*) develops in the liver of the infected rodents. The life cycle is completed when the cats consume infected rodents (laboratory and wild) or any other intermediate host [1-8]. The parasite is of zoonotic significance and human beings can act as accidental intermediate host [9,10]. *Hymenolepis diminuta*, the “rat tapeworm” is also a zoonotic parasite [11-14].

Its occurrence has been considered rare in laboratory rodents. Its epidemiology involves primarily wild rodents and even primates. Intermediate hosts include beetles (*Tribolium confusum*, *Tenebrio molitor*), moths or fleas (*Nosopsyllus fasciatus*) [2,3,6,8,15]. The infection with both parasites in rodents is considered generally asymptomatic, with rare complications [1-3,5,16-19]. It is recommended to practice depopulation of the infected colonies for the control of both parasites [3]. Both parasites had been reported from Mizoram [20-21].

Here, we report the investigation of mortality in wistar rats associated with co-infection of *C. fasciolaris* and *H. diminuta* and its management with common anthelmintics.

## Materials and Methods

### Ethical approval

There was no sacrifice or animal experimentation involved in the present study therefore approval from Institutional Animal Ethics Committee was not required. However, there was no deviation from rules of ethical treatment to animals during the study.

### Animals

Mortality was observed in 9 out of 25 male rats of age between 3 and 5 months kept in the laboratory animal house of Department of Pharmacology and Toxicology College of Veterinary Sciences & A.H., Central Agricultural University, Selesih, Aizawl for research purpose. Post-mortem was conducted on all the 9 rats found dead in their cages during a period of 7 days.

### Clinical examination

The live rats of affected colonies were observed for external signs of disease for a month from the commencement of mortality, considered as day-1 (Table-1). Fecal samples from the affected colonies were examined every 3^rd^ day by preparing direct fecal smear from the day-1.

**Table-1:** Mortality pattern, diagnosis and treatment during the course of mortality

Day	Number of Rats dead	Treatment given	Number of Rats infected with *C. fasciolaris*	Number of Rats infected with *H. diminuta*
1	1	Not given	1	1
2	2	Not given	1	2
3	3	*Fentas Plus*^®^	2	3
4	1	*Fentas Plus*^®^	1	1
5	1	Not given	1	1
6	0	Not given	0	0
7	1	Not given	1	1

C. fasciolaris=Cysticercus fasciolaris, H. diminuta=Hymenolepis diminuta

### Post-mortem examination and sample collection

A thorough and scientific necropsy was conducted of the dead rats and all important findings were photographed. The livers showing parasitic cysts were removed and collected in normal saline solution warmed up to 37ºC. Morphology of the larvae was studied for their identification after dissecting the capsules from the freshly collected samples [2]. The gastrointestinal tracts were examined and the tapeworms as well as the intestinal contents were collected for detecting the presence of parasitic ova by preparing direct fecal smears. For histopathological studies representative tissue samples with parasitic cyst *in-situ* were preserved in 10% neutral buffered formalin.

### Histopathological Studies

Tissue samples were embedded in paraffin wax and 4-5µm thick sections were cut for routine hematoxylin and eosin staining [22]. Observations were recorded by photomicrography.

## Results and discussion

### Clinical and post-mortem findings

**Figure-1:**
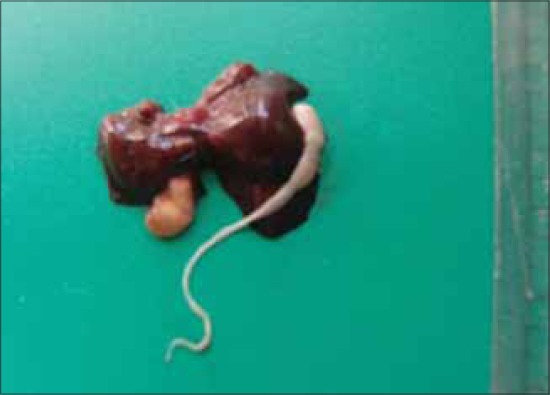
Liver of Wistar rat with a *Cysticercus fasciolaris* dissected out of a cyst.

**Figure-2:**
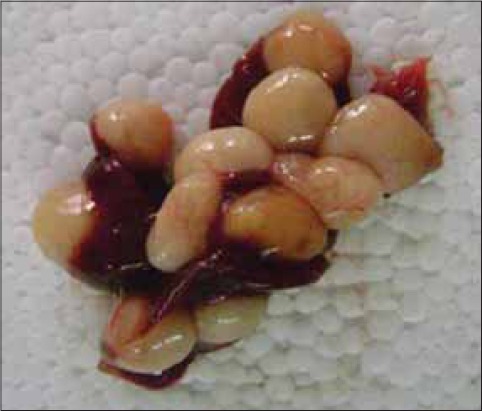
Liver of Wistar rat with 11 cysts of *Cysticercus fasciolaris*.

The affected rats were grossly weak, dull, with rough hair coat and pale mucus membranes. Liver of 7 out of 9 dead rats revealed white cysts about 7-10 mm in diameter embedded in their parenchyma (Table-1, Figures-1 and 2). The cysts had a well-defined capsule wall. The number of cysts observed varied from 1 to as many as 11 in a liver (Figure-2). Variable number of parasitic cysts indicate that the infestation was gradually increasing due to continuous exposure to eggs of *T. taeniaeformis*. On dissecting the cysts, white colored and segmented *strobilocercii* were found coiled inside (Figure-1). They were around 60-80 mm in length and the scolices were large with two rows of rosettlar hooks. The segmented strobila lacked genital organs. The morphology of the *strobilocercii* and their anatomical site of predilection in rats confirmed them to be *C. fasciolaris* [2-3].

**Figure-3:**
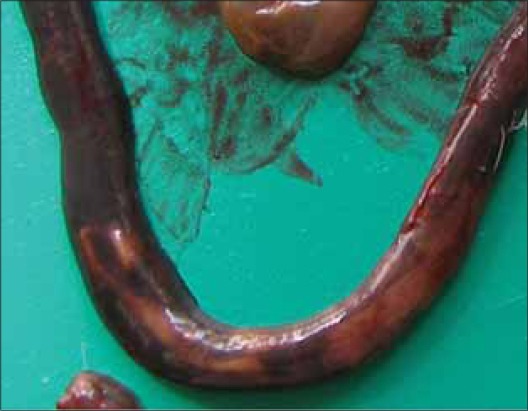
Adult tapeworm (*Hymenolepis diminuta*) visible through the serosa of intestine of rat.

**Figure-4:**
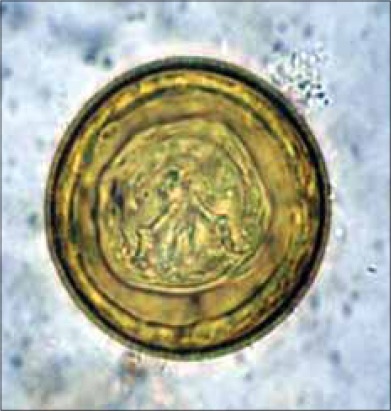
Egg of *Hymenolepis diminuta* (×1000)

Adult tapeworms were also found in the intestines of all 9 dead rats (Table-1). There was thinning of the intestinal wall, and the tapeworm could be located from the serosal surface of the intestines (Figure-3). They were around 50 mm long and 3-4 mm wide. The scolices were pear shaped, unarmed and bore suckers. At the most only 2 or 3 scolices could be recovered from a rat. The eggs were spherical, embryonated with three pairs of small hooks (Figure-4). The morphological characters of worms and eggs and its site of predilection confirmed the species as *H. diminuta* [2-3]. The affected rats revealed severe hemorrhagic enteritis with catarrhal and blood tinged intestinal contents in almost all portions of intestines (Figure-3). Apart from mild to moderate the grossly visible congestion, other visceral organs revealed no specific pathological changes. Random sampling of fecal pellets from the rat colonies also revealed the eggs of *H. diminuta* on fecal smear examination. Mild infections with *H. diminuta* have been considered non-pathogenic [23]. However, it had been reported that anemia and disturbance in liver function can occur in *H. diminuta* infection in rats [19]. The immune system of the rat can control the number of *H. diminuta* in its intestines [16], which may be a reason of finding only few parasites in the studied rats. Emaciation and catarrhal hemorrhagic enteritis were a consistent finding in the dead rats. A heavy infection with this parasite can cause increased intestinal permeability with other pathological changes varying from catarrhal enteritis to chronic enterocolitis. Thus the normal physiology of the infected host is disturbed [3].

### Histopathology

**Figure-5:**
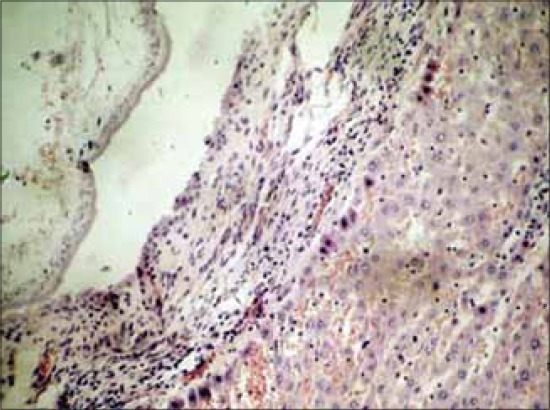
Proliferation of fibrous connective tissue with infiltration of mononuclear cells in adjoining parenchyma of liver to *Cysticercus fasciolaris* cysts (H and E, ×400).

**Figure-6:**
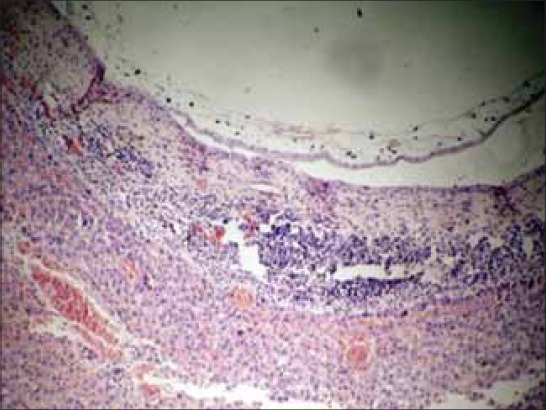
Severe vascular engorgement and proliferation of fibrous connective tissue with infiltration of mononuclear cells in adjoining parenchyma of liver to *Cysticercus fasciolaris* cysts (H and E, ×400).

Histopathology of affected portion of the liver revealed cross section of parasite surrounded by a developing capsule of fibrous connective tissue, a zone of infiltrating inflammatory cells. The fibrous connective tissue comprised of loose collagen fibers and some fibroblasts. The inflammatory zone comprised of extensive infiltration of neutrophils, Kupffer cells and a few epitheloid cells in some foci (Figures-5 and 6). The blood capillaries in the hepatic parenchyma proximal to the capsule revealed extensive engorgement (Figure-6). Similar findings have been reported by other researchers [5,24]. The hepatocytes around the affected area were unremarkable and apparently normal. It is, therefore, possible that if infection is low the affected host may seem clinically normal as generally accepted [1-3]. However, there are few reports of association of parasitic cysts with development of neoplasms [5,17,18,25].

### Treatment and Control

**Figure-7:**
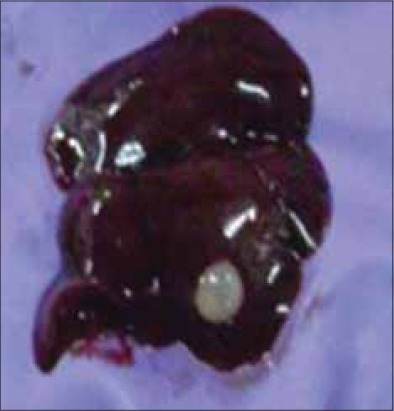
A reduced cyst found in the liver of a rat which died after treatment with anthelmintics.

The remaining rats (16 out of 25) were treated with *Fentas Plus*^®^ (Fenbendazole-150 mg + Praziquantel-50 mg) diluted in 25 ml of distilled water and given at the rate of 30 and 10 mg/kg body weight according to standard recommended therapy [26]. Treatment was given on day 3 and 4 (Table-1) since the commencement of mortality. The stored litter and feed were discarded and replaced with a new lot. Only two rats which were already showing severe illness died post-treatment (Table-1). Two weeks after the treatment a rat, which died due to accidental trauma, revealed a small capsulated cyst in the liver on post-mortem examination (Figure-7). It was smaller in size, about 3-4 mm in diameter, as compared to previously observed ones and did not contain the *strobilocercus*. The intestines were also free of tapeworms. The periodic examination of fecal smears also did not reveal any tapeworm eggs after the treatment with anthelmintics. Individually both parasites are not known to cause acute mortality at the observed level. The co-infection with both helminths seems to have resulted in the observed disease condition and mortality in affected rats. Involvement of secondary bacterial infection also seems likely. However, as there was no antibiotic treatment given and the mortality stopped after anthelmintic administration and hence, it seems most probable that the bacteria involved were probably opportunistic pathogens.

## Conclusion

To our knowledge this is the first report of mortality at remarkable level associated with co-infection of *C. fasciolaris* and *H. diminuta* and its treatment and control with common anthelmintics, from north eastern hilly region of India. According to the findings of present study it is concluded that co-infection of *C. fasciolaris* and *H. diminuta* can cause mortality in wistar rats. It was also concluded that the mortality and the infection can be controlled with common anthelmintics. The study opens scope for avoiding depopulation and replacement of rodent colonies especially in North Eastern Hilly region of India where such a practice can be expensive and time consuming. Considering the epidemiological aspect, it can be indirectly concluded that life cycle stage of *T. taeniaeformis* exist in local cats and possibly other wild felids and rodents. The rodents in the region also harbor *H. diminuta*. There is definite evidence of contamination of laboratory animal house with cat feces and infected insect intermediate hosts of *H. diminuta*. Since both the parasites have zoonotic significance, their potential public health hazard in the region is emphasized by the present study. The study highlights the need for control measures required for breaking the life cycle of the parasites, strict hygiene and biosecurity measures in the laboratory houses.

## Authors’ contributions

YDS conducted the study and data recording. RSA analyzed the data. Manuscript was drafted and revised by RSA under the guidance of YDS. Both authors read and approved the final manuscript.

## References

[1] Jithendran K.P, Somvanshi R (1998). Experimental infection of mice with *Taenia taeniaeformis* eggs from cats - Course of infection and pathological studies. Indian J. Exp. Biol.

[2] Soulsby E.J.L (2005). Cestodes. In: Helminths, arthropods and protozoa of domesticated animals.

[3] Baker D.G (2007). Parasites of rats and mice. In *Flynn's* parasites of laboratory animals.

[4] Singla L.D, Aulakh G.S, Sharma R, Juyal P.D, Singh J (2009). Concurrent infection of *Taenia taeniaeformis* and *Isospora felis* in a stray kitten: a case report. Vet. Med.

[5] Karim A.J (2010). Scanning electron microscopy and histological morphology of *Cysticercus fasciolaris* which induced fibrosarcomas in laboratory rats. Ann. Microsc.

[6] Kataranovski M, Zolotarevski L, Belij S, Mirkov I, Stošić J, Popov A, Kataranovski D (2010). First record of *Calodium hepaticum* and *Taenia taeniaeformis* liver infection in wild Norway rats (*Rattus norvegicus*) in Serbia. Arch. Biol. Sci.

[7] Rodríguez-Vivas R.I, Panti-May J.A, Parada-López J, Hernández-Betancourt S.F, Ruiz-Piña H.A (2011). The occurrence of the larval cestode *Cysticercus fasciolaris* in rodent populations from the Cuxtal ecological reserve, Yucatan, Mexico. J. Helminthol.

[8] Pakdel N, Naem S, Rezaei F, Chalehchaleh A.A (2013). A survey on helminthic infection in mice (*Mus musculus*) and rats (*Rattus norvegicus* and *Rattus rattus*) in Kermanshah, Iran. Vet. Res. Forum.

[9] Miyazaki I (1991). Helminthic Zoonoses.

[10] Ekanayake S, Warnasuriya N.D, Samarakoon P.S, Abewickrama H, Kuruppuarachchi N.D, Dissanaike A.S (1999). An unusual 'infection'of a child in Sri Lanka with *Taenia taeniaeformis* of the cat. Ann Trop. Med. Parasitol.

[11] Mowlavi G, Mobedi I, Mamishi S, Rezaeian M, HaghiAshtiani M.T, Kashi M (2008). *Hymenolepis diminuta* (Rodolphi 1819) infection in a child from Iran. Iran. J. Publ. Health.

[12] Karuna T, Khadanga S (2013). A case of *Hymenolepis diminuta* in a young male from Odisha. Trop Parasitol.

[13] Kołodziej P, Rzymowska J, Stępień-Rukasz H, Lorencowicz R, Lucińska M, Dzióbek M (2014). Analysis of a child infected with *Hymenolepis diminuta* in Poland. Ann. Agri. Environ. Med.

[14] Tiwari S, Karuna T, Rautaraya B (2014). *Hymenolepis diminuta* infection in a child from a rural area: A rare case report. J. Lab. Physician.

[15] Makki M.S, Shahbazi F, Teimoori S, Rokni M.B, Abaei M.R, Mobedi I, Hassanpour G, Mowlavi G (2011). Establishment of *Hymenolepis diminuta* life cycle to provide parasite mass production. Iran. J. Parasitol.

[16] Andreassen J, Hopkins C.A (1980). Immunologically mediated rejection of *Hymenolepis diminuta* by its normal host, the rat. J. Parasitol.

[17] Kohn D.F, Barthold S.W, Fox J.G, Cohn B.J, Loew F.M (1984). Biology and diseases of rats. Laboratory animal medicine.

[18] Hanes M.A (1995). Fibrosarcomas in two rats arising from hepatic cysts of *Cysticercus fasciolaris*. Vet. Pathol.

[19] Goswami R, Singh S.M, Kataria M, Somvanshi R (2011). Clinicopathological studies on spontaneous *Hymenolepis diminuta* infection in wild and laboratory rats. Braz. J. Vet. Pathol.

[20] Malsawmtluangi C, Tandon V (2009). Helminth parasite spectrum in rodent hosts from bamboo growing areas of Mizoram, North-east India. J. Parasit. Dis.

[21] Malsawmtluangi C, Prasad P.K, Biswal D.K, Tandon V (2011). Morphological and molecular identification of the metacestode parasitizing the liver of rodent hosts in bamboo growing areas of Mizoram, northeast India. Bioinformation.

[22] Luna L.G (1968). Manual of Histologic Staining Methods of the AFIP.

[23] Insler G.D, Roberts L.S (1976). *Hymenolepis diminuta*: lack of pathogenecity in the healthy rat host. Exp. Parasitol.

[24] Moudgil A.D, Singla L.D, Gupta K, Daundkar P.S, Vemu B (2014). Histopathological and morphological studies on natural Cysticercus fasciolaris infection in liver of Wistar rats. J. Parasit Dis.

[25] Irizarry-Rovira A.R, Wolf A, Bolek M (2007). *Taenia taeniaeformis* induced metastatic hepatic sarcoma in a pet rat (*Rattus norvegicus*). J. Exotic Pet Med.

[26] McKellar Q.A (1989). Drug dosages for small mammals. In Pract.

